# Supplementary material for: Dydrogesterone as an oral alternative to vaginal progesterone for IVF luteal phase support: A systematic review and individual participant data meta-analysis
Source: PLoS One. 2020 Nov 4;15(11):e0241044. doi: 10.1371/journal.pone.0241044 (PMC7641447; doi:10.1371/journal.pone.0241044)
Supplement: S1 File — (DOCX) [file pone.0241044.s001.docx]

# Dydrogesterone as an oral alternative to vaginal progesterone for IVF luteal phase support: A systematic review and individual participant data meta-analysis

**Short title**: Dydrogesterone versus MVP: IPD meta-analysis

Georg Griesinger^1,^*, Christophe Blockeel^2^, Elke Kahler^3^, Claire Pexman-Fieth^4^, Jan I. Olofsson^5,6^, Stefan Driessen^7^, Herman Tournaye^2^

^1^ Department of Gynecological Endocrinology and Reproductive Medicine, University Hospital of Schleswig-Holstein, Lübeck, Germany

^2^ Center for Reproductive Medicine, Universitair Ziekenhuis Brussel, Brussels, Belgium

^3^ Global Biometrics, Established Pharmaceuticals Division, Abbott Laboratories GmbH, Hannover, Germany

^4^ Global Clinical Development, Established Pharmaceuticals Division, Abbott GmbH, Wiesbaden, Germany

^5^ Global Medical Affairs, Established Pharmaceuticals Division, Abbott Products Operations AG, Allschwil, Switzerland

^6^ Division of Obstetrics and Gynecology, Department of Women’s and Children’s Health, Karolinska Institutet, Stockholm, Sweden

^7^ Global Biometrics, Established Pharmaceuticals Division, Abbott Healthcare Products BV, Weesp, The Netherlands

*****Corresponding author

E‑mail: [georg.griesinger@uni-luebeck.de](mailto:georg.griesinger@uni-luebeck.de) (GG)

# Supporting information

S1 Fig 1. Fixed effect and random effects model meta-analysis of (A) risk difference and (B) odds ratio for pregnancy rate from aggregate data from Lotus I and II (oral dydrogesterone versus MVP).


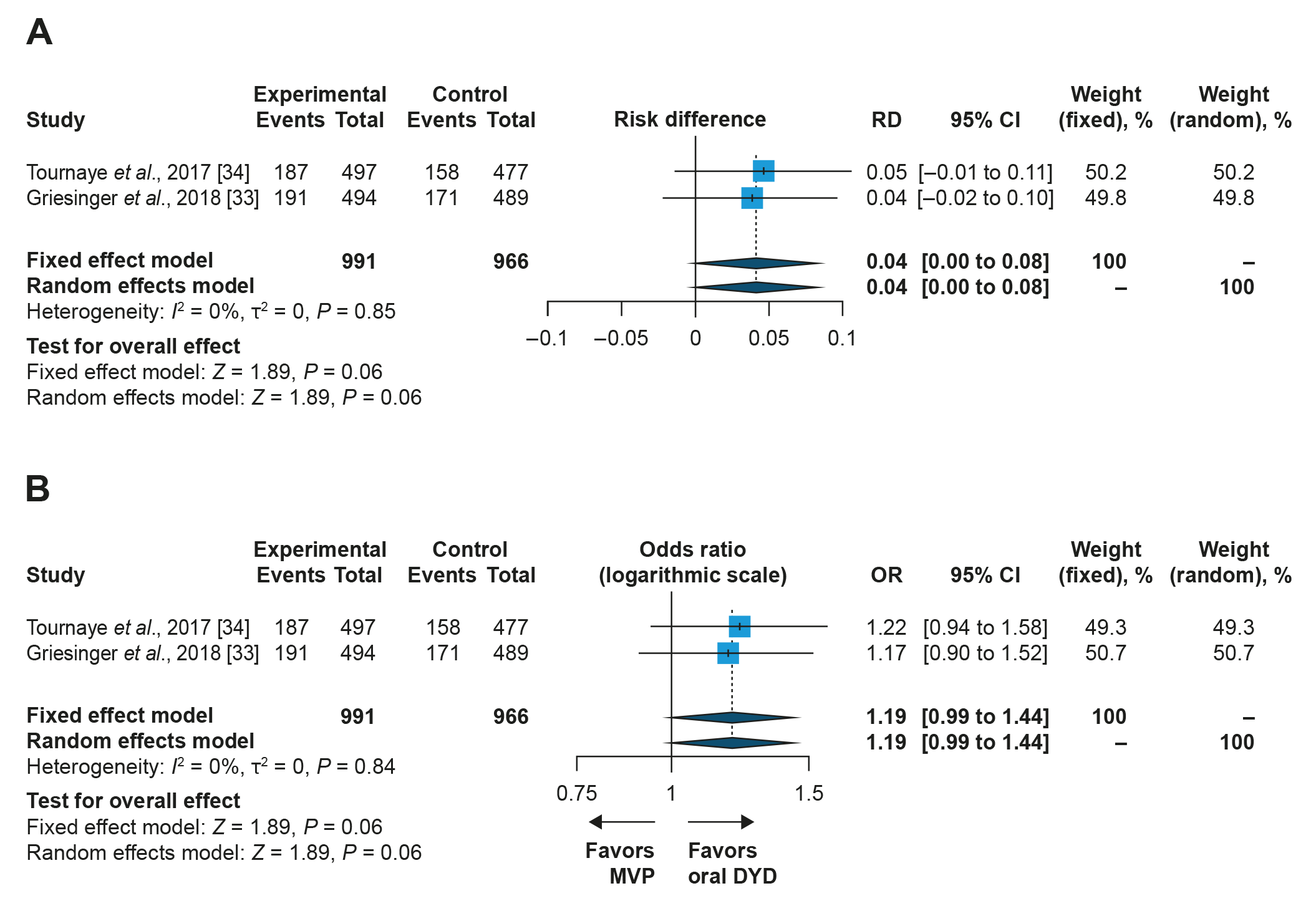


CI, confidence interval; DYD, dydrogesterone; MVP, micronized vaginal progesterone; OR, odds ratio; RD, risk difference.

S2 Fig 2. Fixed effect and random effects model meta-analysis of (A) risk difference and (B) odds ratio for live birth rate from aggregate data from Lotus I and II (oral dydrogesterone versus MVP).


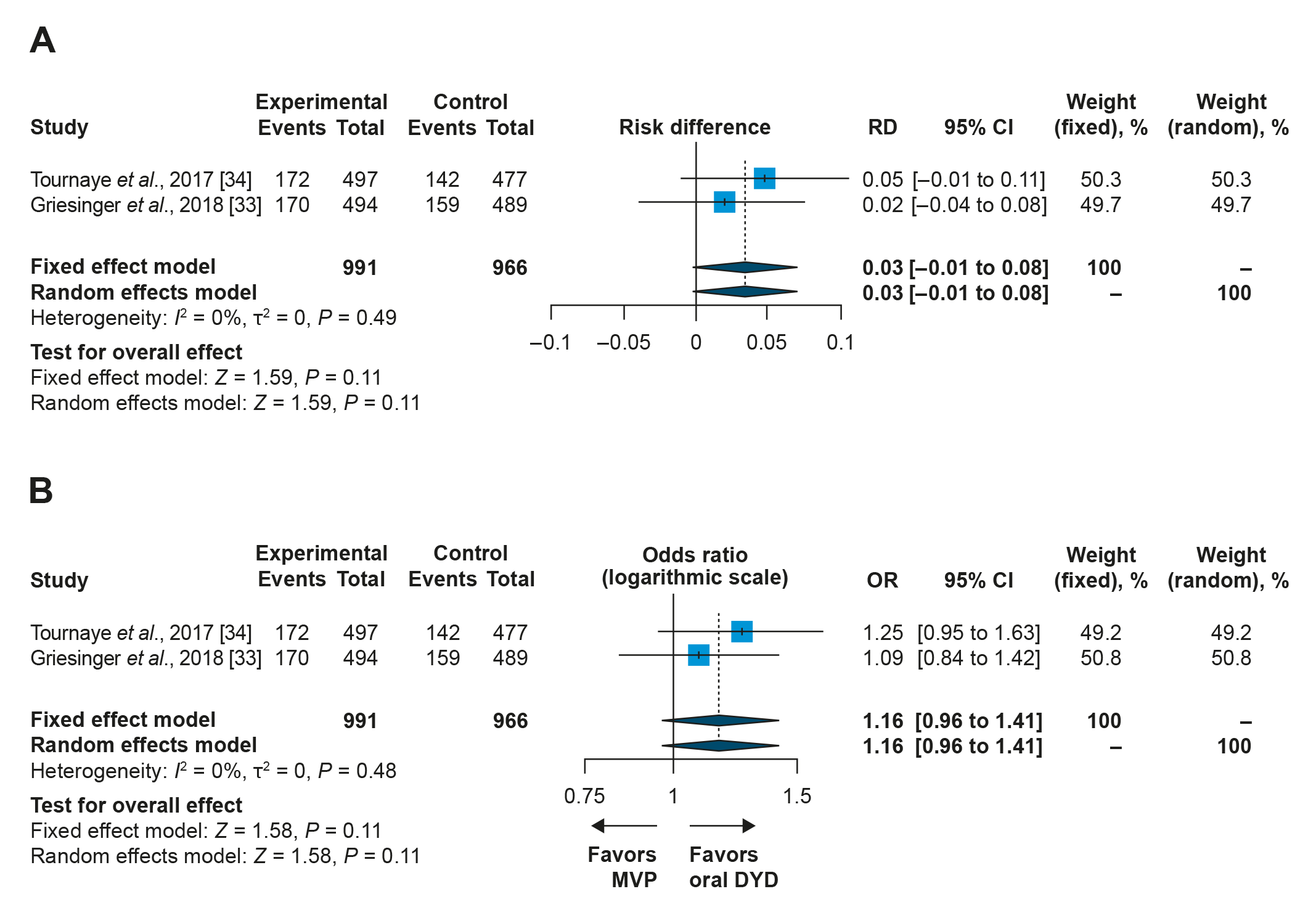
CI, confidence interval; DYD, dydrogesterone; MVP, micronized vaginal progesterone; OR, odds ratio; RD, risk difference.

S3 Fig 3. Fixed effect and random effects model meta-analysis of (A) risk difference and (B) odds ratio for pregnancy rate from aggregate data (oral dydrogesterone versus MVP).


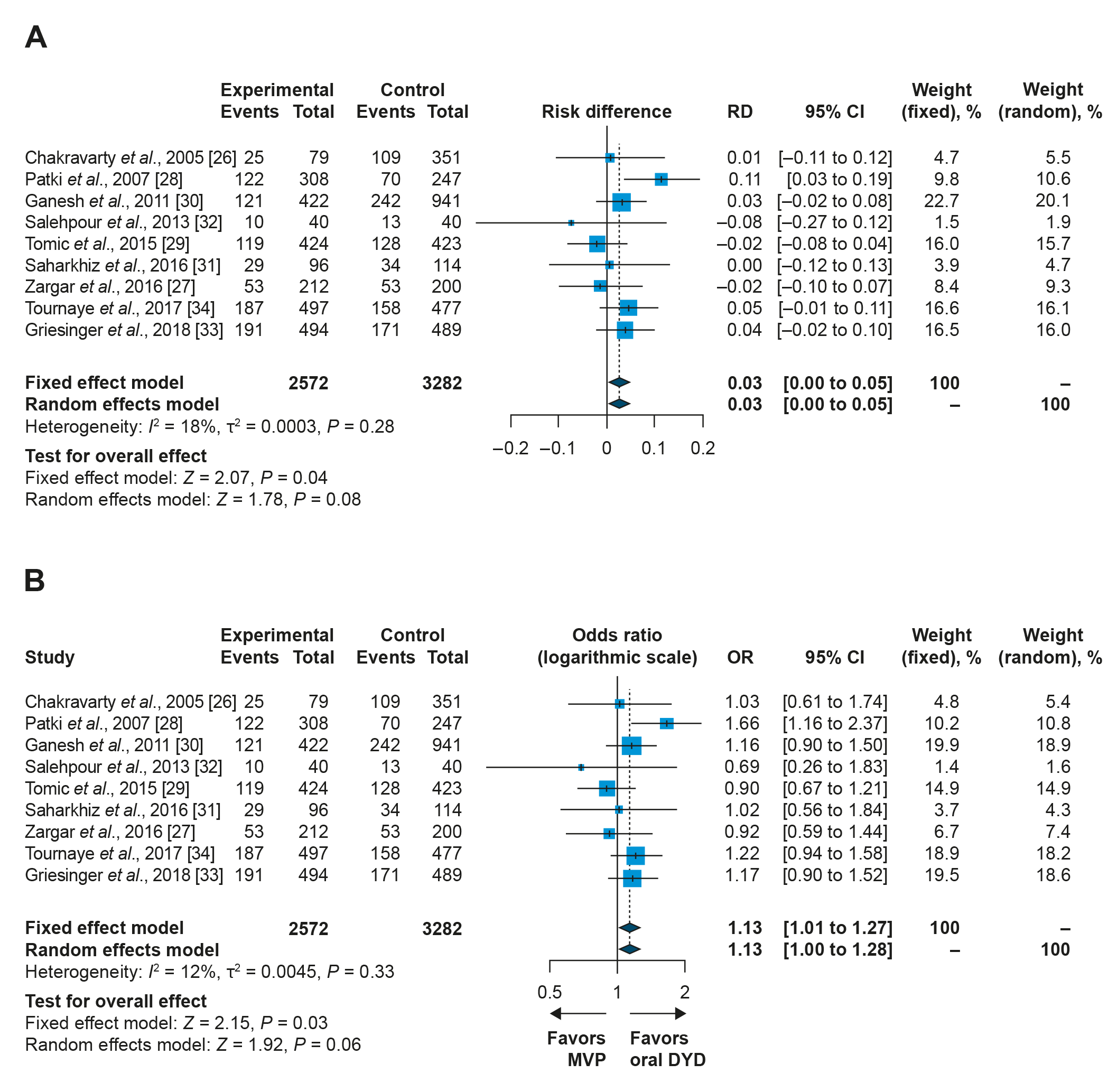


CI, confidence interval; DYD, dydrogesterone; MVP, micronized vaginal progesterone; OR, odds ratio; RD, risk difference.

S4 Fig 4. Fixed effect and random effects model meta-analysis of (A) risk difference and (B) odds ratio for live birth rate from aggregate data (oral dydrogesterone versus MVP).


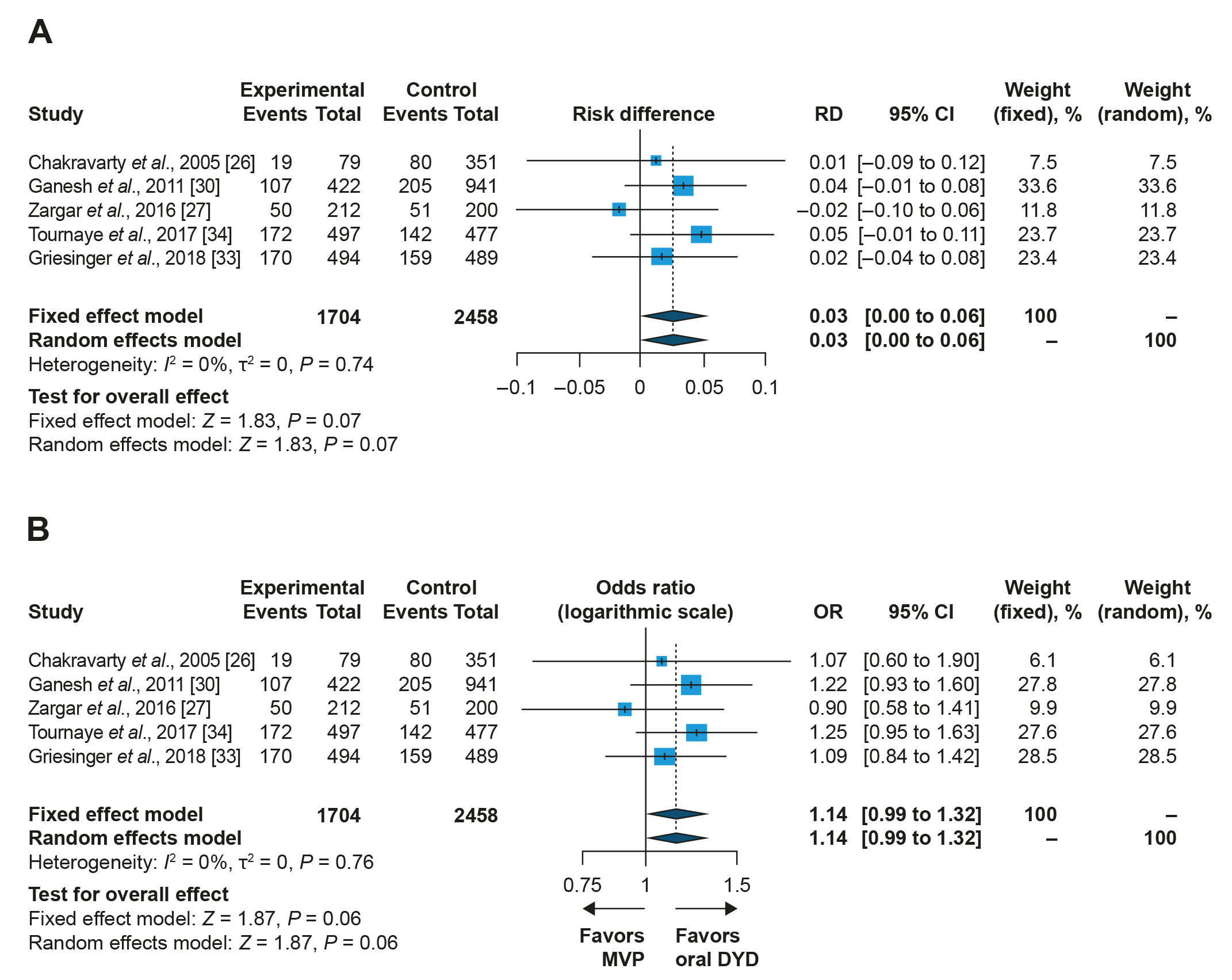
CI, confidence interval; DYD, dydrogesterone; MVP, micronized vaginal progesterone; OR, odds ratio; RD, risk difference.

S1 Table. Potential predictor variables identified and included in the IPD stepwise logistic regression analysis.

| Variable | Live birth rate | Ongoing pregnancy rate^a^ |
| --- | --- | --- |
| Age | *P* = 0.0009 | *P* < 0.0001 |
| Country | *P* < 0.0001 | *P* < 0.0001 |
| Study site | *P* < 0.0001 | *P* < 0.0001 |
| BMI | *P* = 0.30 | *P* = 0.21 |
| Day of embryo transfer | *P* < 0.0001 | *P* < 0.0001 |
| Number of embryos transferred | *P* = 0.30 | *P* = 0.46 |
| Treatment | *P* = 0.115 | *P* = 0.060 |
| ICSI | *P* = 0.0177 | *P* = 0.0073 |

BMI, body mass index; ICSI, intracytoplasmic sperm injection.

Note: *P*-values are derived from simple logistic regression with each respective variable as a single factor and was the starting point for the stepwise selection procedure. As country is a linear combination of the sites, it was not included in the model; the number of embryos transferred was kept in the model for both pregnancy rate and live birth rate.

^a^12 weeks of gestation.

S2 Table. Summary of analyses.

|  | **Statistic** | **IPD** | **AD** | **IPD and AD** |
| --- | --- | --- | --- | --- |
| *Pregnancy rate* |  |  |  |  |
| Two studies^a^ | OR | 1.32 | 1.19 | NA |
|  | 95% CI | 1.08 to 1.61 | 0.99 to 1.44 | NA |
|  | *P-*value | 0.0075 | 0.06^b^ | NA |
|  | Figure presented | Fig. 3 | S1B Fig | NA |
| All nine studies^c^ | OR | NA | 1.13 | 1.16 |
|  | 95% CI | NA | 1.00 to 1.28 | 1.01 to 1.34 |
|  | *P*-value | NA | 0.06^b^ | 0.04^b^ |
|  | Figure presented | NA | S3B Fig | Fig. 4A |
| *Live birth rate* |  |  |  |  |
| Two studies^a^ | OR | 1.28 | 1.16 | NA |
|  | 95% CI | 1.04 to 1.57 | 0.96 to 1.41 | NA |
|  | *P*-value | 0.0214 | 0.11^b^ | NA |
|  | Figure presented | Fig. 3 | S2B Fig | NA |
| All nine studies^c^ | OR | NA | 1.14 | 1.19 |
|  | 95% CI | NA | 0.99 to 1.32 | 1.03 to 1.38 |
|  | *P*-value | NA | 0.06^b^ | 0.02^b^ |
|  | Figure presented | NA | S4B Fig | Fig. 4B |

AD, aggregate data; CI, confidence interval; IPD, individual participant data; NA, not applicable; OR, odds ratio.

^a^Tournaye *et al*. (2017) [34]; Griesinger *et al*. (2018) [33].

^b^Random effects model.

^c^Chakravarty *et al*. (2005) [26]; Patki *et al.* (2007) [28]; Ganesh *et al.* (2011) [30]; Salehpour *et al.* (2013) [32]; Tomic *et al*. (2015) [29]; Saharkhiz *et al*. (2016) [31]; Zargar *et al*. (2016) [27]; Tournaye *et al*. (2017) [34]; Griesinger *et al*. (2018) [33].

S3 Table. Meta-analysis of IPD: influence of potential predictor variables on live birth rate (FAS).^a^

| Variable | Parameter | | Live birth | | OR (95% CI) | *P*-value |
| --- | --- | --- | --- | --- | --- | --- |
|  |  |  | **Yes** | **No** |  |  |
| *Significant predictor variables*^b^ | | | | | | |
| Treatment | Oral DYD | *n*/*N* (%) | 342/991 (34.5) | 649/991 (65.5) | Oral DYD vs MVP: 1.28 (1.04 to 1.57) | *P* = 0.0214 |
|  | MVP | *n*/*N* (%) | 301/966 (31.2) | 665/966 (68.8) |  |  |
| Age, years | *N*  Mean (SD) | | 643  31.6 (4.3) | 1314  32.4 (4.6) | 0.96 (0.94 to 0.99) | *P* = 0.0032 |
| Study site | NA^c^ | | | | | *P* < 0.0001 |
| Day of embryo transfer | < Day 5 | *n*/*N* (%) | 386/643 (60.0) | 900/1314 (68.5) | ≥ Day 5 vs < Day 5:  1.27 (1.12 to 1.44) | *P* = 0.0002 |
|  | ≥ Day 5 | *n*/*N* (%) | 257/643 (40.0) | 414/1314 (31.5) |  |  |
| *Non-significant variable* | | | | | | |
| Number of embryos transferred | 0 | *n*/*N* (%) | 0/643 | 3/1314 (0.2) | > 2 vs 1: 0.5 (0.14 to 1.81)  2 vs 1: 1.27 (0.99 to 1.65) |  |
|  | 1 | *n*/*N* (%) | 242/643 (37.6) | 513/1314 (39.0) |  | *P* = 0.065 |
|  | 2 | *n*/*N* (%) | 398/643 (61.9) | 780/1314 (59.4) |  |  |
|  | > 2 | *n*/*N* (%) | 3/643 (0.5) | 18/1314 (1.4) |  |  |

CI, confidence interval; DYD, dydrogesterone; FAS, full analysis sample; IPD, individual participant data; MVP, micronized vaginal progesterone; NA, not applicable; OR, odds ratio; SD, standard deviation**.**

^a^Three subjects in the oral dydrogesterone group were discontinued prior to embryo transfer due to study drug‑related issues; these subjects were included in the FAS as failures (not pregnant).

^b^ORs, 95% CIs, and *P*-values were calculated by logistic regression analysis for all variables included in the final model of the stepwise selection procedure.

^c^75 sites in the dataset.

S4 Table. Maternal adverse events reported by ≥ 2% of subjects (pooled IPD from two studies; safety sample).

| Adverse event, *n* (%) | Oral DYD (*N* = 1036) | MVP (*N* = 1023) | All (*N* = 2059) |
| --- | --- | --- | --- |
| Vaginal hemorrhage^a^ | 120 (11.6) | 97 (9.5) | 217 (10.5) |
| Miscarriage (not induced abortion)^b^ | 87 (8.4) | 105 (10.3) | 192 (9.3) |
| Abdominal pain^c^ | 73 (7.0) | 79 (7.7) | 152 (7.4) |
| Nausea | 60 (5.8) | 42 (4.1) | 102 (5.0) |
| Procedural pain | 56 (5.4) | 58 (5.7) | 114 (5.5) |
| Migraine/headache | 47 (4.5) | 50 (4.9) | 97 (4.7) |
| Vomiting^d^ | 45 (4.3) | 38 (3.7) | 83 (4.0) |
| Ovarian hyperstimulation syndrome | 38 (3.7) | 38 (3.7) | 76 (3.7) |
| Anemia^e^ | 34 (3.3) | 27 (2.6) | 61 (3.0) |

DYD, dydrogesterone; IPD, individual participant data; MedDRA, Medical Dictionary for Regulatory Activities; MVP, micronized vaginal progesterone.

These terms included the following preferred terms from MedDRA version 20.1:

^a^Vaginal hemorrhage, hemorrhage in pregnancy, uterine hemorrhage, genital hemorrhage, cervix hemorrhage.

^b^Abortion spontaneous, abortion missed, vanishing twin syndrome, anembryonic gestation, abortion, abortion complete, abortion early, abortion late, imminent abortion, biochemical pregnancy.

^c^Abdominal pain, abdominal pain lower, abdominal pain upper, abdominal discomfort.

^d^Vomiting in pregnancy, vomiting, regurgitation.

^e^Anemia, anemia of pregnancy, iron deficiency anemia, hemoglobin decreased.

S5 Table. Newborn characteristics and adverse events reported in ≥2% of newborns in the two studies (FAS).

| Category | Oral DYD | MVP | Total |
| --- | --- | --- | --- |
| Total number of newborns, n | 418 | 348 | 766 |
| Term newborns, n (%)^a^ (≥37 weeks of gestation) | 299 (71.5) | 262 (75.3) | 561 (73.2) |
| Preterm newborns, n (%)^a^ (> 22 and < 37 weeks of gestation) | 119 (28.5) | 86 (24.7) | 205 (26.8) |
| Weight, kg (mean ± SD) | 2.9 ± 0.7 | 3.0 ± 0.7 | 3.0 ± 0.7 |
| Singleton births, kg (mean ± SD) | 3.2 ± 0.6 | 3.2 ± 0.5 | 3.2 ± 0.5 |
| Multiple births, kg (mean ± SD) | 2.4 ± 0.5 | 2.3 ± 0.6 | 2.4 ± 0.5 |
| Low birth weight (< 2500 g), n (%)^a,b^ | 111 (26.6) | 68 (19.5) | 179 (23.4) |
| Term newborns, n (%)^c^ (≥ 37 weeks of gestation) | 33 (29.7) | 12 (17.6) | 45 (25.1) |
| Term singleton newborns, n (%)^c^ (≥ 37 weeks of gestation) | 7 (6.3) | 5 (7.4) | 12 (6.7) |
| Term multiple (twins/triplets) newborns, n (%)^c^ (≥ 37 weeks of gestation) | 26 (23.4) | 7 (10.3) | 33 (18.4) |
| Preterm newborns, n (%)^c^  (> 22 and < 37 weeks of gestation) | 78 (70.2) | 56 (82.4) | 134 (74.9) |
| Preterm singleton newborns, n (%)^c^ (> 22 and < 37 weeks of gestation) | 18 (16.2) | 12 (17.6) | 30 (16.7) |
| Preterm multiple (twins/triplets) newborns, n (%)^c^ (> 22 and < 37 weeks of gestation) | 60 (54.1) | 44 (64.7) | 104 (58.1) |
| Number of newborns with an adverse event, n (%) | 43 (10.3) | 41 (11.8) | 84 (11.0) |
| Neonatal jaundice^d^ | 12 (2.9) | 10 (2.9) | 22 (2.9) |
| Neonatal respiratory distress syndrome^e^ | 10 (2.4) | 11 (3.2) | 21 (2.7) |

DYD, dydrogesterone; FAS, full analysis sample; MedDRA, Medical Dictionary for Regulatory Activities; MVP, micronized vaginal progesterone; SD, standard deviation.

^a^Percentages calculated according to the number divided by all newborns.

^b^Low birth weight according to birth record and not adverse events.

^c^Percentages calculated according to the number divided by all low birth weight newborns.

^d^Included the following preferred terms from MedDRA Version 20.1: jaundice neonatal, hyperbilirubinemia, hyperbilirubinemia neonatal.

^e^Included the following preferred terms from MedDRA Version 20.1: neonatal respiratory distress syndrome, acute respiratory distress syndrome.

Note: See S6 Table for all congenital, familial, and genetic disorders identified in 35 pregnancies comprising 24 newborns and 11 selective terminations due to malformations.

S6 Table. Congenital, familial, and genetic disorders identified in live newborns and selective terminations due to malformations (pooled IPD from two studies).

| Congenital, familial, and genetic disorders, *n* | Oral DYD  (*N* = 19) | MVP  (*N* = 16) | All  (*N* = 35) |
| --- | --- | --- | --- |
| Newborn | 14 | 10 | 24 |
| Selective terminations due to malformation | 5 | 6 | 11 |
| **Adverse event*** |  |  |  |
| Atrial septal defect^a^ | 5 | 7 | 12 |
| Heart disease congenital^a^ | 2 | 4 | 6 |
| Patent ductus arteriosus^a^ | 1 | 4 | 5 |
| Trisomy 21 | 1 | 2 | 3 |
| Turner syndrome | 2 | 0 | 2 |
| Ventricular septal defect^a^ | 2 | 0 | 2 |
| Interruption of aortic arch^a^ | 1 | 0 | 1 |
| Accessory auricle | 1 | 0 | 1 |
| Amniotic band syndrome | 1 | 0 | 1 |
| Congenital central nervous system anomaly | 1 | 0 | 1 |
| Congenital cystic kidney disease | 1 | 0 | 1 |
| Congenital hand malformation | 1 | 1 | 2 |
| Cystic lymphangioma | 1 | 0 | 1 |
| Intestinal malrotation | 1 | 0 | 1 |
| Kinematic imbalances due to suboccipital strain | 1 | 0 | 1 |
| Renal dysplasia | 1 | 0 | 1 |
| Talipes | 1 | 0 | 1 |
| Tracheoesopheal fistula | 1 | 0 | 1 |
| Congenital aortic anomaly^a^ | 0 | 1 | 1 |
| Congenital hydrocephalus | 0 | 1 | 1 |
| Congenital tricuspid valve atresia^a^ | 0 | 1 | 1 |
| Kidney malformation | 0 | 1 | 1 |
| Pulmonary artery atresia^a^ | 0 | 1 | 1 |
|  |  |  |  |
| Spina bifida | 0 | 1 | 1 |
| Trisomy 13 | 0 | 1 | 1 |
| Univentricular heart^a^ | 0 | 1 | 1 |

* Each newborn or fetus could have more than one AE.

DYD, dydrogesterone; IPD, individual participant data; MedDRA, Medical Dictionary for Regulatory Activities; MVP, micronized vaginal progesterone; PT, preferred term.

The terms shown are the PTs from MedDRA version 20.1:

^a^The PT grouping ‘congenital heart disease’ occurred in 8 cases in the oral dydrogesterone group and 11 cases in the MVP group as some of the newborns/fetuses had more than one congenital cardiac-related adverse event. It included the following nine MedDRA PTs: atrial septal defect, heart disease congenital, patent ductus arteriosus, ventricular septal defect, interruption of aortic arch, congenital aortic anomaly, congenital tricuspid valve atresia, pulmonary artery atresia, univentricular heart.

S1 Checklist. PRISMA checklist.


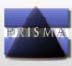
**PRISMA 2009 Checklist**

| **Section/topic** | **#** | **Checklist item** | **Reported on page #** |
| --- | --- | --- | --- |
| **TITLE** | | |  |
| Title | 1 | Identify the report as a systematic review, meta-analysis, or both. | Page 1 |
| **ABSTRACT** | | |  |
| Structured summary | 2 | Provide a structured summary including, as applicable: background; objectives; data sources; study eligibility criteria, participants, and interventions; study appraisal and synthesis methods; results; limitations; conclusions and implications of key findings; systematic review registration number. | Page 2 |
| **INTRODUCTION** | | |  |
| Rationale | 3 | Describe the rationale for the review in the context of what is already known. | Pages 3-5 |
| Objectives | 4 | Provide an explicit statement of questions being addressed with reference to participants, interventions, comparisons, outcomes, and study design (PICOS). | Pages 4-5 |
| **METHODS** | | |  |
| Protocol and registration | 5 | Indicate if a review protocol exists, if and where it can be accessed (e.g., Web address), and, if available, provide registration information including registration number. | Page 5 |
| Eligibility criteria | 6 | Specify study characteristics (e.g., PICOS, length of follow-up) and report characteristics (e.g., years considered, language, publication status) used as criteria for eligibility, giving rationale. | Pages 6-8 |
| Information sources | 7 | Describe all information sources (e.g., databases with dates of coverage, contact with study authors to identify additional studies) in the search and date last searched. | Pages 6 |
| Search | 8 | Present full electronic search strategy for at least one database, including any limits used, such that it could be repeated. | Pages 6-7 |
| Study selection | 9 | State the process for selecting studies (i.e., screening, eligibility, included in systematic review, and, if applicable, included in the meta-analysis). | Pages 6-7 |
| Data collection process | 10 | Describe method of data extraction from reports (e.g., piloted forms, independently, in duplicate) and any processes for obtaining and confirming data from investigators. | Page 7 |
| Data items | 11 | List and define all variables for which data were sought (e.g., PICOS, funding sources) and any assumptions and simplifications made. | Page 7 |
| Risk of bias in individual studies | 12 | Describe methods used for assessing risk of bias of individual studies (including specification of whether this was done at the study or outcome level), and how this information is to be used in any data synthesis. | Page 9 |
| Summary measures | 13 | State the principal summary measures (e.g., risk ratio, difference in means). | Pages 7-8 |
| Synthesis of results | 14 | Describe the methods of handling data and combining results of studies, if done, including measures of consistency (e.g., I^2^) for each meta-analysis. | Pages 7-8 |
