# Supplementary material for: Dydrogesterone as an oral alternative to vaginal progesterone for IVF luteal phase support: A systematic review and individual participant data meta-analysis
Source: PLoS One. 2020 Nov 4;15(11):e0241044. doi: 10.1371/journal.pone.0241044 (PMC7641447; doi:10.1371/journal.pone.0241044)
Supplement: Attachment — Submitted filename: review pone.docx [file pone.0241044.s002.docx]

This is a well written manuscript that presents interesting results related to one of the most controversial and fascinating aspects of IVF treatments: the support of the luteal phase. The work describes a method of meta-analysis (IPD), which could be not so familiar to several readers. However, the strengths and limitations of the method are well described. The results may have important clinical implications.

I would suggest some changes to the authors:

In the abstract, only the IPD results are mentioned. It would be better also to include the results of the general meta-analysis with aggregated data in the abstract.

Introduction, line 60

The authors wrote “MVP is associated with its own vaginal administration-related side effects, including irritation and discharge”. It would be interesting to know whether vaginal administration is associated with an increased risk of bacterial vaginitis / alterations in the female microbiota that can lead to unfavorable reproductive outcomes. This would strengthen the rationale for proposing an alternative route of administration.

This aspect should be detailed also in the discussion, lines 537-544.

Introduction, line 98-103

It is not usual to anticipate the results in the introduction. I would recommend to remove the paragraph and leave it to the results / conclusions.

Analysis, line 160-161

Please explain to the reader what the choice of significance at 0.3 and 0.35 is based on

Table 1

The studies included in the meta-analysis have different methods of progesterone administration. Notably, the first day of progesterone administration differs between the studies. The same applies to the total weeks of therapy. Authors should discuss whether there is evidence that such variability may impact the validity of the luteal phase support and possibly recognize it as a study limitation.

Table 2

Subjects who had at least one newborn: the percentage value seems to be missing

Obstetric outcome and fetal/newborn adverse events-line 392

Oral DYD is associated with a higher number of preterm deliveries and low birth weight. Has it been checked whether this difference is significant? How do the authors would explain this observation?

Discussion

The discussion on the methodological and statistical part is extensively developed. However, only at the end of the conclusion an aspect that could be very interesting to the readers is discussed: why does oral progesterone determine a greater chance of success? What are the pharmacological mechanisms underlying this difference? Are there any innovative theories or recent evidence that can show how vaginal administration can have fewer positive effects? Please discuss,

Discussion line 421-459

The authors comment on the impact of age, blastocyst transfer, treatment site, number of embryos transferred, BMI, and use of ICSI as potential “prognostic factors”. This aspect is sufficiently well known among the readers and can be developed more lightly. The aspect that should be developed is not whether these factors have an impact on IVF treatment, but rather whether these factors have been associated with different outcomes because of the luteal phase support strategy. Is there evidence in this sense?

What is important is reported in lines 436-437: there are no differences in the two study groups under comparison for age, blastocyst transfer, treatment site. What about number of embryos transferred, BMI, and use of ICSI?

Discussion, lines 460-463.

The authors admit the lack of some data on patients' baseline characteristics and on the treatment protocol / intermediate outcome. Again, authors should discuss whether there are interactions with the modality of the luteal phase support. For example, can the authors reasonably assert that oral progesterone administration is equally valid in agonist or antagonist cycles? What about the trigger mode? In specific cases, such as induction with agonist and fresh transfer with support of the luteal phase with low doses of hCG, is there a rationale that can make the oral route preferable over the vaginal one or vice versa?

In brief, please discuss any interactions between prognostic factors and luteal phase support, not between prognostic factors and IVF in general.

Discussion line 464

In the paragraph relating to safety outcomes, explain whether the association of preterm labor and low birth weight with oral progesterone has a rationale and should concern clinicians.
