# Supplementary material for: Dydrogesterone as an oral alternative to vaginal progesterone for IVF luteal phase support: A systematic review and individual participant data meta-analysis
Source: PLoS One. 2020 Nov 4;15(11):e0241044. doi: 10.1371/journal.pone.0241044 (PMC7641447; doi:10.1371/journal.pone.0241044)
Supplement: Attachment — Submitted filename: Griesinger et al_Response to Reviewers.docx [file pone.0241044.s003.docx]

UNIVERSITÄTSKLINIKUM

Schleswig-Holstein

**Campus Lübeck**

### Telefon Fax

### Sekretariat Prof. Griesinger 0451/505-778 212 -299

### Anmeldung Kinderwunsch: 0451/505778-10 -299

**Homepage:** www.uksh.de/Kinderwunsch_Luebeck

**Email**: kinderwunsch@uksh.de

**Homepage PID**: [www.uksh.de/pid-luebeck](about:blank)

**E-Mail:**	pid-luebeck@uksh.de

**Datum:** 24.09.20

**Sektion für gynäkologische Endokrinologie und Reproduktionsmedizin**

Leitung: Prof. Dr. med. Georg Griesinger, *M.Sc*.

UNIVERSITÄTSKLINIKUM Schleswig-Holstein

Sektion für Reproduktionsmedizin Ratzeburger Allee 160 23538 Lübeck

**Auskunft erteilt:** Prof. Dr. med. univ. Georg Griesinger

Joerg Heber

Editor-in-Chief

PLOS ONE

per electronic submission

Dear Dr Heber,

On behalf of my co-authors, I am pleased to enclose the manuscript ‘*Dydrogesterone as an oral alternative to vaginal progesterone for IVF luteal phase support: A systematic review and individual participant data meta-analysis*’ for resubmission to *PLOS ONE*.

We thank the reviewers for their insightful comments and believe that these have now been fully addressed, where possible, in this revised version. Please find detailed responses to each comment in the table at the end of this letter. The alterations we have made within the manuscript have also been clearly denoted by tracked changes in the enclosed revised version.

Further to the comment regarding the competing interests section, we were happy to amend this. Please see below the updated statement, with the new sentence added (as requested):

G.G.'s institution has received investigator fees from Abbott during the conduct of the Lotus I and Lotus II studies. Outside of this submitted work, G.G. has received nonfinancial support from MSD, Ferring, Merck Serono, IBSA, Finox, TEVA, Glycotope and Gedeon Richter, as well as personal fees from MSD, Ferring, Merck Serono, IBSA, Finox, TEVA, Glycotope, Vitrolife, NMC Healthcare, ReprodWissen, Biosilu, Gedeon Richter and ZIVA. C.B.’s institution has received investigator fees from Abbott during the conduct of the Lotus I and Lotus II studies. C.B. is the President of the Belgian Society of Reproductive Medicine (unpaid) and Section Editor of Reproductive BioMedicine Online. C.B. has received grants from Ferring, participated in an MSD-sponsored trial and has received consultancy fees from Ferring, MSD, BioMérieux, Abbott and Merck. E.K. is an employee of Abbott Laboratories GmbH, Hannover, Germany and owns shares of Abbott. C.P.-F. is an employee of Abbott GmbH & Co. KG, Wiesbaden, Germany and owns shares in Abbott. J.I.O. is an employee of Abbott Products Operations AG, Allschwil, Switzerland.

S.D. is an employee of Abbott Healthcare Products BV, Weesp, The Netherlands and owns shares in Abbott. H.T.'s institution has received investigator fees from Abbott during the conduct of the Lotus I and Lotus II studies. H.T.’s institution has received grants from Merck, MSD, Goodlife, Cook, Roche, CooperSurgical, Besins, Ferring and Allergan. H.T. has received consultancy fees from Gedeon Richter, Merck, Ferring, Abbott and ObsEva. This does not alter our adherence to PLOS ONE policies on sharing data and materials.

We hope that the manuscript now meets the requirements of *PLOS ONE* and I look forward to hearing from you at your earliest convenience.

Yours sincerely,

Prof. Dr. med. Georg Griesinger, M.Sc
Email: [georg.griesinger@uni-luebeck.de](mailto:georg.griesinger@uni-luebeck.de)

|  | **Reviewer comments** | **Response (**The text was **added** or ~~deleted~~**)** |
| --- | --- | --- |
| **Review comments within the email** | | |
|  | We note that the flowchart you included (Figure 1) does not include the reasons you excluded studies from analysis. Please revise the figure to include the reasons for exclusion. Please also revise the figure to include how many studies were excluded for each reason. | The flowchart (Figure 1) has been updated as requested    Fig 1. Flow diagram of the process of selecting and excluding studies for the meta-analyses.  IPD, individual participant data; IVF, in vitro fertilization. ~~aInclusion criteria not met.~~ ~~Excluded articles included those reporting frozen thawed IVF cycles, studies that did not report the prespecified outcome measures of interest, non prospective studies, and conference abstracts.~~  Study selection (tacked change document, lines: 156-161  Excluded articles included ~~those reporting frozen thawed IVF cycles,~~ studies that did not report the prespecified outcome measures of interest, **review articles, retrospective** ~~non-prospective~~ studies, **case reports,** and conference abstracts. The full texts of the remaining articles were examined in detail to determine their suitability for inclusion. **All i**~~i~~nvestigators of studies that met the inclusion criteria (see Eligibility Criteria section) were contacted to request access to IPD.  ***New text***  Excluded articles included studies that did not report the prespecified outcome measures of interest, review articles, retrospective studies, case reports, and conference abstracts. The full texts of the remaining articles were examined in detail to determine their suitability for inclusion. All investigators of studies that met the inclusion criteria (see Eligibility Criteria section) were contacted to request access to IPD. |
|  | Amend to Competing Interests statement | See updated statement within the letter, as requested |
|  | The gold standard for progesterone luteal phase supplementation is PIO (progesterone in oil). In the introduction, the authors should state why they chose to study vaginal administration vs oral instead of oral vs PIO | Firstly, from a regulatory perspective, micronized vaginal progesterone is the standard of care on a global scale, as there is no progesterone-in-oil licensed for use in the context of assisted reproduction in most countries, including US, UK and Germany (to name a few). Of note, both LOTUS trials were registration trials.  Secondly, for registration studies, double-blind, randomized controlled studies are performed against other products that are registered for the desired indication. The sponsor of the LOTUS I and II trials does not consider it ethically possible to compare oral tablets against intramuscular product (progesterone in oil) in a double-blind fashion.  No text added to the manuscript |
|  | All figures/tables should be at the end of the manuscript, instead of being inserted in the middle | The manuscript was structured as stated here: <https://journals.plos.org/plosone/s/submission-guidelines>  **Figures:** ‘*Do not include figures in the main manuscript file. Each figure must be prepared and submitted as an individual file*.’  **Tables:** ‘*Place each table in your manuscript file directly after the paragraph in which it is first cited (read order). Do not submit your tables in separate files’*  No changes have been made, as the guidelines were followed |
|  | There are several different doses of oral progesterone used in the various studies. Why was this done and did this change any of the findings? | **Study characteristics and risk of bias (tracked document, lines: 252-259)**  Characteristics of all nine eligible studies are shown in Table 1, and S2 Table provides an overall summary of the IPD, aggregate data, and combined IPD and aggregate data meta-analyses conducted in this study. **There were several different doses of oral dydrogesterone used in the studies. Four studies prescribed 30 mg/day [27, 28, 33, 34]; three studies prescribed 20 mg/day for luteal phase support, and this was in line with the registered dosing for “treatment of infertility due to luteal insufficiency” [26, 29, 30]. The two studies that prescribed 40 mg/day also prescribed a higher dose of MVP [31, 32]. We do not think that the dosage differences influence the findings of this analysis, with its main conclusion on route of administration and drug type, and not dosage.** In five of the nine eligible studies, luteal phase support was commenced on the day of oocyte retrieval and continued until 12 weeks of gestation [29, 31-34].  ***New text***  Characteristics of all nine eligible studies are shown in Table 1, and S2 Table provides an overall summary of the IPD, aggregate data, and combined IPD and aggregate data meta-analyses conducted in this study. There were several different doses of oral dydrogesterone used in the studies. Four studies prescribed 30 mg/day [27, 28, 33, 34]; three studies prescribed 20 mg/day for luteal phase support, and this was in line with the registered dosing for “treatment of infertility due to luteal insufficiency” [26, 29, 30]. The two studies that prescribed 40 mg/day also prescribed a higher dose of MVP [31, 32]. We do not think that the dosage differences influence the findings of this analysis, with its main conclusion on route of administration and drug type, and not dosage. The authors do not think that the dosage differences influence the findings of this analysis, with its main conclusion on route of administration and drug type and not dosage. In five of the nine eligible studies, luteal phase support was commenced on the day of oocyte retrieval and continued until 12 weeks of gestation [29, 31-34]. |
| **Review comments within the Word Document** | | |
| 1 | In the abstract, only the IPD results are mentioned. It would be better also to include the results of the general meta-analysis with aggregated data in the abstract. | **Abstract (tracked document, lines: 39-42)**  In the meta-analysis of IPD, oral dydrogesterone was associated with a significantly higher chance of ongoing pregnancy at 12 weeks of gestation (odds ratio [OR], 1.32; 95% confidence interval [CI], 1.08 to 1.61; P = 0.0075) and live birth (OR, 1.28; 95% CI, 1.04 to 1.57; P = 0.0214) compared to MVP. **A meta-analysis combining IPD and aggregate data for all nine studies also demonstrated a statistically significant difference between oral dydrogesterone and MVP (pregnancy: OR, 1.16; 95% CI, 1.01 to 1.34; P = 0.04; live birth: OR, 1.19; 95% CI, 1.03 to 1.38; P = 0.02).**  ***New text***  In the meta-analysis of IPD, oral dydrogesterone was associated with a significantly higher chance of ongoing pregnancy at 12 weeks of gestation (odds ratio [OR], 1.32; 95% confidence interval [CI], 1.08 to 1.61; P = 0.0075) and live birth (OR, 1.28; 95% CI, 1.04 to 1.57; P = 0.0214) compared to MVP. A meta-analysis combining IPD and aggregate data for all nine studies also demonstrated a statistically significant difference between oral dydrogesterone and MVP (pregnancy: OR, 1.16; 95% CI, 1.01 to 1.34; P = 0.04; live birth: OR, 1.19; 95% CI, 1.03 to 1.38; P = 0.02). |
| 2a | Introduction  The authors wrote “MVP is associated with its own vaginal administration-related side effects, including irritation and discharge”. It would be interesting to know whether vaginal administration is associated with an increased risk of bacterial vaginitis / alterations in the female microbiota that can lead to unfavorable reproductive outcomes. This would strengthen the rationale for proposing an alternative route of administration.  Discussion  This aspect should be detailed also in the discussion, lines 537-544 | **Introduction (tracked document, lines: 67-81)**  However, MVP is associated with its own vaginal administration related side effects, including irritation and discharge [14, 15], and some women may not be comfortable using MVP due to cultural reasons [9]. **Recently, MVP has been shown in a pilot IVF study to affect the local microbiome [16]. It has been postulated, following endometrial alterations in the uterine microbiome in response to inflammation, that a progesterone-resistant endometrium can develop [17].**  **Dydrogesterone is a stereoisomer of progesterone, where the hydrogen atom at carbon 9 is in the β position and the methyl group at carbon 10 is in the α position, the reverse of the progesterone structure (hence denoted “retro” progesterone) [18].** **In addition, there is an additional double bond between carbon 6 and 7 whereby the flat steroid configuration is modified, creating a “bent” conformation with enhanced rigidity compared to progesterone [19, 20]. This is thought to account for dydrogesterone having a high selectivity for progesterone receptors with potent progestogenic activity but no or negligible agonistic activity at androgen, glucocorticoid, and mineralocorticoid receptors [21]. In contrast to progesterone, dydrogesterone has higher oral bioavailability [22], which, together with its activity and high specificity for progesterone receptors, along with the efficacy of oral dydrogesterone at a relatively low dose, may minimize side effects [18]**.  ***New text***  However, MVP is associated with its own vaginal administration related side effects, including irritation and discharge [14, 15], and some women may not be comfortable using MVP due to cultural reasons [9]. Recently, MVP has been shown in a pilot IVF study to affect the local microbiome [16]. Recently, MVP has been shown in a pilot IVF study to affect the local microbiome [16]. It has been postulated, following endometrial alterations in the uterine microbiome in response to inflammation, that a progesterone-resistant endometrium can develop [17].  Dydrogesterone is a stereoisomer of progesterone, where the hydrogen atom at carbon 9 is in the β position and the methyl group at carbon 10 is in the α position, the reverse of the progesterone structure (hence denoted “retro” progesterone) [18]. In addition, there is an additional double bond between carbon 6 and 7 whereby the flat steroid configuration is modified, creating a “bent” conformation with enhanced rigidity compared to progesterone [19, 20]. This is thought to account for dydrogesterone having a high selectivity for progesterone receptors with potent progestogenic activity but no or negligible agonistic activity at androgen, glucocorticoid, and mineralocorticoid receptors [21]. In contrast to progesterone, dydrogesterone has higher oral bioavailability [22], which, together with its activity and high specificity for progesterone receptors, along with the efficacy of oral dydrogesterone at a relatively low dose, may minimize side effects [18].  **Discussion (tracked document, lines: 612-617)**  Although MVP may be the most prescribed route of luteal phase support administration [7], it may not be the most preferred route of administration since women often find vaginal preparations less appealing than oral administration, particularly due to comfort issues [58]. Importantly, improvement in interdisciplinary scientific efforts may be required to increase our understanding of patient needs and preferences [60].  **Earlier, we put forward a hypothesis that MVP concentrations in the vagina may alter the local microbiota, which has become a recent focus of interest in the context of IVF [23,62]. To address this question, some of the authors herein are presently conducting a prospective, randomized, double-blind, double-dummy, two-arm, cross-over study in healthy oocyte donor volunteers to assess if there are differences in vaginal microbiota between groups taking oral dydrogesterone or MVP [63].**  ***New text***  Although MVP may be the most prescribed route of luteal phase support administration [7], it may not be the most preferred route of administration since women often find vaginal preparations less appealing than oral administration, particularly due to comfort issues [60]. Importantly, improvement in interdisciplinary scientific efforts may be required to increase our understanding of patient needs and preferences [61].  Earlier, we put forward a hypothesis that MVP concentrations in the vagina may alter the local microbiota, which has become a recent focus of interest in the context of IVF [23,62]. To address this question, some of the authors herein are presently conducting a prospective, randomized, double-blind, double-dummy, two-arm, cross-over study in healthy oocyte donor volunteers to assess if there are differences in vaginal microbiota between groups taking oral dydrogesterone or MVP [63]. |
| 3 | Introduction, line 98-103  It is not usual to anticipate the results in the introduction. I would recommend to remove the paragraph and leave it to the results / conclusions. | **Introduction (tracked document, lines: 117-123)**  In summary, the aim of this study was to systematically and comprehensively collate the existing evidence on the efficacy and safety of oral dydrogesterone versus MVP when used as luteal phase support in IVF. ~~The analysis identified that oral dydrogesterone was associated with a significantly higher ongoing pregnancy rate and live birth rate than MVP administered as capsules or as a gel. The meta-analysis of IPD indicated that per 1000 women treated with oral dydrogesterone versus MVP, 381 and 314 achieved an ongoing pregnancy, respectively. This finding has implications for the standard of care in IVF treatment but also highlights the necessity of further research into existing progestogens and administration routes.~~  ***New text***  In summary, the aim of this study was to systematically and comprehensively collate the existing evidence on the efficacy and safety of oral dydrogesterone versus MVP when used as luteal phase support in IVF.  **Discussion (tracked document, lines: 637-645)**  **Our analysis identified that oral dydrogesterone was associated with a significantly higher ongoing pregnancy rate and live birth rate than MVP administered as capsules or as a gel. The meta-analysis of IPD indicated that per 1000 women treated with oral dydrogesterone versus MVP, 381 and 314 achieved an ongoing pregnancy, respectively. This finding is postulated to have implications for the standard of care in IVF treatment.** Given the widespread use of MVP and the large number of IVF/ICSI cycles performed worldwide, further research into **existing progestogens and administration routes for** luteal phase support is warranted, where patient acceptability is taken into consideration in addition to efficacy and safety.  ***New text***  Our analysis identified that oral dydrogesterone was associated with a significantly higher ongoing pregnancy rate and live birth rate than MVP administered as capsules or as a gel. The meta-analysis of IPD indicated that per 1000 women treated with oral dydrogesterone versus MVP, 381 and 314 achieved an ongoing pregnancy, respectively. This finding is postulated to have implications for the standard of care in IVF treatment. Given the widespread use of MVP and the large number of IVF/ICSI cycles performed worldwide, further research into existing progestogens and administration routes for luteal phase support is warranted, where patient acceptability is taken into consideration in addition to efficacy and safety. |
| 4 | Analysis and summary measures IPD  Analysis, line 160-161  Please explain to the reader what the choice of significance at 0.3 and 0.35 is based on. | There is no fixed rule for these cutoff probabilities. However, reasonable boundaries need to be chosen in order to be able to add and remove variables in the stepwise selection process. This section in the manuscript was rewritten for better clarity. The following text **(in bold)** was added or ~~deleted~~  **Analysis and summary measures IPD** (tracked document, lines: 180-186)  The following rule was applied **for the logistic regression model with a stepwise selection:** if **a** ~~this~~ comparison resulted in a ~~significance level~~ **cut off probability** of ≤ 0.3, then the prognostic factor was included in ~~a logistic regression~~ **the** model ~~with stepwise selection~~; **the** **cut off probability for removing factors from the model was set at** ~~of~~ 0.35 ~~was required for a potential prognostic factor to stay in the model~~. **Study s~~S~~**site (not country) was chosen to be included in the model as these were highly correlated (collinearity).  ***New text***  The following rule was applied for the logistic regression model with a stepwise selection: if a comparison resulted in a cutoff probability of ≤ 0.3, then the prognostic factor was included in the model; the cutoff probability for removing factors from the model was set at 0.35. Study site (not country) was chosen to be included in the model as these were highly correlated (collinearity). |
| 5 | Table 1 Study characteristics and risk of bias  The studies included in the meta-analysis have different methods of progesterone administration. Notably, the first day of progesterone administration differs between the studies. The same applies to the total weeks of therapy. Authors should discuss whether there is evidence that such variability may impact the validity of the luteal phase support and possibly recognize it as a study limitation. | Study characteristics and risk of bias (tracked document, lines: 259-265)  **In five of the nine eligible studies, luteal phase support was commenced on the day of oocyte retrieval and continued until 12 weeks of gestation [29, 31-34]. In the study by Patki et al. [28], luteal phase support was also started on the day of oocyte retrieval; however, the duration of treatment was unclear. In two studies, it was started on the day of embryo transfer and continued until 12 weeks of gestation [26, 30]. In the study by Zargar et al. [27], there was no documentation regarding the day luteal phase support was commenced, but treatment was continued until 12 weeks of gestation.**  *New text*  In five of the nine eligible studies, luteal phase support was commenced on the day of oocyte retrieval and continued until 12 weeks of gestation [29, 31-34]. In the study by Patki et al. [28], luteal phase support was also started on the day of oocyte retrieval; however, the duration of treatment was unclear. In two studies, it was started on the day of embryo transfer and continued until 12 weeks of gestation [26, 30]. In the study by Zargar et al. [27], there was no documentation regarding the day luteal phase support was commenced, but treatment was continued until 12 weeks of gestation.  Discussion (tracked document, lines: 554-564).  A recent systematic review and meta-analysis concluded that intramuscular progestogen administration for luteal phase support in fresh-cycle IVF provided greater clinical benefit versus other routes of administration [54]. In that meta-analysis, it was reported that the mean pregnancy rates increased from 14.7% for untreated women to 30.7%, 36.4%, 36.6%, and 44.0% after oral, vaginal, subcutaneous, and intramuscular progestogen supplementation, respectively [54]. **Not surprisingly, the optimal time to achieve higher clinical pregnancy rates is to commence luteal phase support between oocyte retrieval and embryo transfer (OR, 1.31), with oocyte retrieval +1 day reported as being most beneficial. Similarly, clinical pregnancy rates were found to be equivalent when progestogen supplementation was given during a period between ≤ 3 weeks and up to 12 weeks (OR, 1.06) of gestation [54]. This is in line with findings from eight of the nine studies included in the present meta-analysis.** ~~However, it~~It is important to note that a binomial logistic regression model was used **in the recent meta-analysis**, which does not allow for comparisons of treated versus control groups and also does not allow for comparisons of heterogeneity between studies; additionally, both retrospective and non-randomized trials were included [54].  *New text*  A recent systematic review and meta-analysis concluded that intramuscular progestogen administration for luteal phase support in fresh-cycle IVF provided greater clinical benefit versus other routes of administration [54]. In that meta-analysis, it was reported that the mean pregnancy rates increased from 14.7% for untreated women to 30.7%, 36.4%, 36.6%, and 44.0% after oral, vaginal, subcutaneous, and intramuscular progestogen supplementation, respectively [54]. Not surprisingly, the optimal time to achieve higher clinical pregnancy rates is to commence luteal phase support between oocyte retrieval and embryo transfer (OR, 1.31), with oocyte retrieval +1 day reported as being most beneficial. Similarly, clinical pregnancy rates were found to be equivalent when progestogen supplementation was given during a period between ≤ 3 weeks and up to 12 weeks (OR, 1.06) of gestation [54]. This is in line with findings from eight of the nine studies included in the present meta-analysis. It is important to note that a binomial logistic regression model was used in the recent meta-analysis, which does not allow for comparisons of treated versus control groups and also does not allow for comparisons of heterogeneity between studies; additionally, both retrospective and non-randomized trials were included [54]. |
| 6 | Table 2  Subjects who had at least one newborn: the percentage value seems to be missing | Table 2  The percentage should not have been included as it is 100% for all. This the percentage sign has been deleted. The following text was ~~deleted~~:  Subjects who had at least one newborn, n ~~(%)~~^~~b~~^ |
| 8 | Figures: on the basis of which criteria the studies have been sorted in the forest plots (it is generally suggested to order the studies in chronological order or by effect size). | Figures  The figures have been re-arranged to present the included studies in chronological order. Please find new drawings of Figure 4A and Supplementary Figures 3A /3B |
| 8 | Obstetric outcome and fetal/newborn adverse events  Oral DYD is associated with a higher number of preterm deliveries and low birth weight. Has it been checked whether this difference is significant? How do the authors would explain this observation?  Discussion  In the paragraph relating to safety outcomes, explain whether the association of preterm labor and low birth weight with oral progesterone has a rationale and should concern clinicians. | Obstetric outcome and fetal/newborn adverse events: (tracked document, lines: 426-437) Overall, there were 179 newborns with low birth weight (< 2500g), with 26.6% (111/418) and 19.5% (68/348) **of all newborns** occurring in the oral dydrogesterone and MVP groups, respectively (S5 Table). ~~Among these newborns with low birth weight, 134 were preterm,~~ **Not surprisingly, the majority of newborns with low birth weight were detected among preterm multiples (twins/triplets) (58.1% [104/179]);** ~~with~~ 70.2% (78/111) and 82.4% (56/68) **of newborns with low birth weight were preterm** ~~occurring~~ in the oral dydrogesterone and MVP groups, respectively (S5 Table). **Among the newborns born at term and with low birth weight, in the dydrogesterone group (29.7% [33/111]) they were predominantly multiples (twins/triplets) compared to singletons (23.4% [26/111] versus 6.3% [7/111], respectively) (S5 Table). For MVP, there were 17.6% (12/68) of newborns with low birth weight born at term, and they were more evenly distributed between multiples (twins/triplets) and singletons (10.3% [7/68] versus 7.4% [5/68], respectively) (S5 Table).** *New text* Overall, there were 179 newborns with low birth weight (< 2500g), with 26.6% (111/418) and 19.5% (68/348) of all newborns occurring in the oral dydrogesterone and MVP groups, respectively (S5 Table). Not surprisingly, the majority of newborns with low birth weight were detected among preterm multiples (twins/triplets) (58.1% [104/179]); 70.2% (78/111) and 82.4% (56/68) of newborns with low birth weight were preterm in the oral dydrogesterone and MVP groups, respectively (S5 Table). Among the newborns born at term and with low birth weight, in the dydrogesterone group (29.7% [33/111]) they were predominantly multiples (twins/triplets) compared to singletons (23.4% [26/111] versus 6.3% [7/111], respectively) (S5 Table). For MVP, there were 17.6% (12/68) of newborns with low birth weight born at term, and they were more evenly distributed between multiples (twins/triplets) and singletons (10.3% [7/68] versus 7.4% [5/68], respectively) (S5 Table). S5 Table. Newborn characteristics and adverse events reported in ≥2% of newborns in the two studies (FAS). Category \| Oral DYD \| MVP \| Total; Total number of newborns, n \| 418 \| 348 \| 766 Low birth weight (<2500 g), n (%)**^a,^**^b^ \| 111 (26.6) \| 68 (19.5) \| 179 (23.4); Term newborns, n (%)^c^ (≥37 weeks of gestation) \| 33 (29.7) \| 12 (17.6) \| 45 (25.1); **Term singleton newborns, n (%)^c^ (≥37 weeks of gestation)** \| **7 (6.3)** \| **5 (7.4)** \| **12 (6.7)**; **Term multiple (twins/triplets) newborns, n (%)^c^ (≥37 weeks of gestation)** \| **26 (23.4)** \| **7 (10.3)** \| **33 (18.4)**; Preterm newborns, n (%)^c^ (>22 and <37 weeks of gestation) \| 78 (70.2) \| 56 (82.4) \| 134 (74.9); **Preterm Singleton newborns, n (%)^c^ (>22 and <37 weeks of gestation)** \| **18 (16.2)** \| **12 (17.6)** \| **30 (16.7)**; **Preterm multiple (twins/triplets) newborns, n (%)^c^ (>22 and <37 weeks of gestation)** \| **60 (54.1)** \| **44 (64,7)** \| **104 (58.1)** ^a^Percentages calculated according to the number divided by all newborns. ^b^Low birth weight according to birth record and not adverse events. ^c^Percentages calculated according to the number divided by all low birth weight newborns. Discussion (tracked document, lines: 507-529) The safety outcomes included in this meta-analysis of IPD (pooled results analyzed using descriptive statistics) were similar between the oral dydrogesterone and MVP groups in both the maternal and fetus/newborn populations. **Specific complications, such as low birth weight and preterm birth, are known to be associated with assisted reproductive technology. In a 2017 review by Kushnir, *et al*. [44], low birth rate was reported in 12.7% of infants (6.7% for singletons following single embryo transfer and 7.3% following double-embryo transfer, 56.3% for twins, and 97.4% for higher-order multiples) and the preterm delivery rate was reported as 16.6% (10.5% for singletons, 67.3% for twins, and 92.3% for higher-order multiples). The practice of multiple embryo transfer remains prevalent in many countries, driven by a focus on maximizing pregnancy rates since early IVF studies showed that single embryo transfer resulted in lower pregnancy rates per transfer compared to double embryo transfer [45, 46]. However, and importantly, the success rates from single embryo transfer has improved drastically in recent years [45, 47]. Due to the increased risk of maternal and fetal complications associated with multiple gestation from double embryo transfer, there has been an increasing worldwide trend in performing single embryo transfer in patients with good prognosis and utilizing surplus embryos in later frozen thawed transfers [44].** **Corresponding to the overall safety outcomes, in the subgroup analysis of** ~~which also applies to~~ congenital heart disease among newborns and selective terminations due to malformations**, similar findings between the oral dydrogesterone and MVP groups in both the maternal and fetus/newborn populations were noted.** This is in **sharp** contrast to a retrospective, case-controlled study from the Gaza region [48] that reported a possible risk for newborn malformations after in utero dydrogesterone exposure. *New text* The safety outcomes included in this meta-analysis of IPD (pooled results analyzed using descriptive statistics) were similar between the oral dydrogesterone and MVP groups in both the maternal and fetus/newborn populations. Specific complications, such as low birth weight and preterm birth, are known to be associated with assisted reproductive technology. In a 2017 review by Kushnir, et al. [44], low birth rate was reported in 12.7% of infants (6.7% for singletons following single embryo transfer and 7.3% following double-embryo transfer, 56.3% for twins, and 97.4% for higher-order multiples) and the preterm delivery rate was reported as 16.6% (10.5% for singletons, 67.3% for twins, and 92.3% for higher-order multiples). The practice of multiple embryo transfer remains prevalent in many countries, driven by a focus on maximizing pregnancy rates since early IVF studies showed that single embryo transfer resulted in lower pregnancy rates per transfer compared to double embryo transfer [45, 46]. However, and importantly, the success rates from single embryo transfer has improved drastically in recent years [45, 47]. Due to the increased risk of maternal and fetal complications associated with multiple gestation from double embryo transfer, there has been an increasing worldwide trend in performing single embryo transfer in patients with good prognosis and utilizing surplus embryos in later frozen thawed transfers [44]. Corresponding to the overall safety outcomes, in the subgroup analysis of congenital heart disease among newborns and selective terminations due to malformations, similar findings between the oral dydrogesterone and MVP groups in both the maternal and fetus/newborn populations were noted. This is in sharp contrast to a retrospective, case-controlled study from the Gaza region [48] that reported a possible risk for newborn malformations after in utero dydrogesterone exposure. |
| 9 | Discussion  The discussion on the methodological and statistical part is extensively developed. However, only at the end of the conclusion an aspect that could be very interesting to the readers is discussed: why does oral progesterone determine a greater chance of success? What are the pharmacological mechanisms underlying this difference? Are there any innovative theories or recent evidence that can show how vaginal administration can have fewer positive effects? Please discuss. | Discussion (tracked document, lines: 632-636)  The underlying reason for vaginal progesterone being suboptimal when compared with an orally active progestogen is not yet understood; however, it may be speculated that it could be due to an insufficient systemic exposure of the patients to the progestogenic compound and/or the specific mode of action of dydrogesterone, **or specific features of dydrogesterone [18].**  *New text*  The underlying reason for vaginal progesterone being suboptimal when compared with an orally active progestogen is not yet understood; however, it may be speculated that it could be due to an insufficient systemic exposure of the patients to the progestogenic compound and/or the specific mode of action of dydrogesterone, or specific features of dydrogesterone [18].  As described earlier in this response, a new paragraph has been added to the introduction to respond to the question.  *New text* (tracked document, lines: 71-81)  **Dydrogesterone is a stereoisomer of progesterone, where the hydrogen atom at carbon 9 is in the β position and the methyl group at carbon 10 is in the α position, the reverse of the progesterone structure (hence denoted “retro” progesterone) [18]. In addition, there is an additional double bond between carbon 6 and 7 whereby the flat steroid configuration is modified, creating a “bent” conformation with enhanced rigidity compared to progesterone [19, 20]. This is thought to account for dydrogesterone having a high selectivity for progesterone receptors with potent progestogenic activity but no or negligible agonistic activity at androgen, glucocorticoid, and mineralocorticoid receptors [21]. In contrast to progesterone, dydrogesterone has higher oral bioavailability [22], which, together with its activity and high specificity for progesterone receptors, along with the efficacy of oral dydrogesterone at a relatively low dose, may minimize side effects [18].** |
| 10 | Discussion line 421-459  The authors comment on the impact of age, blastocyst transfer, treatment site, number of embryos transferred, BMI, and use of ICSI as potential “prognostic factors”.  This aspect is sufficiently well known among the readers and can be developed more lightly.  The aspect that should be developed is not whether these factors have an impact on IVF treatment, but rather whether these factors have been associated with different outcomes because of the luteal phase support strategy. Is there evidence in this sense?  What is important is reported in lines 436-437: there are no differences in the two study groups under comparison for age, blastocyst transfer, treatment site. What about number of embryos transferred, BMI, and use of ICSI? | Discussion (tracked document, lines: 458-498)  The meta-analysis of IPD identified other prognostic factors**, besides luteal phase support treatment,** that influenced the likelihood of ongoing pregnancy and live birth; these included maternal age, day of embryo transfer, and study site. ~~Maternal age is known to be an important factor, with decreasing success from IVF correlating with increasing age , which is in line with the findings of this study. This study also identified that embryo transfer ≥ 5 days after oocyte retrieval (blastocyst stage) was associated with increased likelihood of ongoing pregnancy and live birth compared with transfer < 5 days after oocyte retrieval (cleavage stage). These data are consistent with those reported in a Cochrane systematic review and meta-analysis (low/moderate quality of evidence) , and in line with the recent statement from the American Society for Reproductive Medicine (ASRM) that supports the use of blastocyst transfer in patients with a good prognosis . Finally, IVF success is known to vary according to treatment center , which is in agreement with the findings of this study; this may be due to many factors, including the various protocols, technical proficiency, and population differences .~~ The meta-analysis of IPD also tested for interactions between **luteal phase support** treatment and the other prognostic factors identified**,** but no statistically significant interactions were identified. Thus, the associations of maternal age, day of embryo transfer and study site with clinical outcome were not significantly different between the treatment groups.  In this study**, there were no differences in the number of embryos transferred, BMI, or the use of ICSI between the two groups, and these were no**t ~~the number of embryos transferred, BMI, and use of ICSI were not~~ found to be independent prognostic factors that influenced the likelihood of ongoing pregnancy or live birth. ~~While early IVF studies have shown that single embryo transfer resulted in significantly lower pregnancy rates compared with double embryo transfer , the success rates from single embryo transfer has improved drastically in recent years . Due to the increased risk of maternal and fetal complications associated with multiple gestation from double embryo transfer, there has been an increasing, worldwide trend in performing single embryo transfer in patients with good prognosis and utilizing surplus embryos in later frozen thawed transfers .~~  ~~For this meta-analysis of IPD, BMI, rather than bodyweight, was chosen as a parameter, as it is a more accurate measure of body fat; however, the effect of BMI on pregnancy outcomes from IVF is unclear . The findings from a large retrospective study of fresh IVF cycles suggested that a high BMI is associated with a negative influence on fertility in younger patients, although this effect diminished with increasing age .~~  ~~While ICSI was developed for the most severe forms of male factor infertility, it has rapidly become the most frequently used method of fertilization in assisted reproductive technology . Despite this, existing evidence does not support the preference of ICSI over conventional IVF techniques for couples with non-male factor infertility , with a recent population-based cohort study reporting that ICSI resulted in a similar cumulative live birth rate compared with conventional IVF in this population . As the relative proportion of couples with non-male factor infertility is unknown in this meta analysis, it is not possible to interpret the results in this context~~.  *New text*  The meta-analysis of IPD identified other prognostic factors, besides luteal phase support treatment, that influenced the likelihood of ongoing pregnancy and live birth; these included maternal age, day of embryo transfer, and study site. The meta-analysis of IPD also tested for interactions between luteal phase support treatment and the other prognostic factors identified, but no statistically significant interactions were identified. Thus, the associations of maternal age, day of embryo transfer and study site with clinical outcome were not significantly different between the treatment groups.  In this study, there were no differences in the number of embryos transferred, BMI, or the use of ICSI between the two groups, and these were not found to be independent prognostic factors that influenced the likelihood of ongoing pregnancy or live birth. |
| 11 | Discussion  The authors admit the lack of some data on patients' baseline characteristics and on the treatment protocol / intermediate outcome. Again, authors should discuss whether there are interactions with the modality of the luteal phase support. For example, can the authors reasonably assert that oral progesterone administration is equally valid in agonist or antagonist cycles? What about the trigger mode? In specific cases, such as induction with agonist and fresh transfer with support of the luteal phase with low doses of hCG, is there a rationale that can make the oral route preferable over the vaginal one or vice versa?  In brief, please discuss any interactions between prognostic factors and luteal phase support, not between prognostic factors and IVF in general. | Discussion (tracked document, lines: 499-506)  Other factors that may influence pregnancy outcomes, but were not systematically collected in Lotus I and II, are ovarian reserve [42], ovarian stimulation regimens, gonadotropin releasing hormone agonist or antagonist usage, oocyte maturation trigger, and IVF clinical embryology data [43]. **Based on the non-availability of such study data, any possible interactions between these factors and luteal phase support remain elusive. However, since the Lotus program enrolled over 2000 patients in 14 countries and was shown to be adequately randomized, it may be assumed that it reflects the general clinical practice of fresh-cycle IVF, where both agonist and antagonist cycles were represented.**  *New text*  Other factors that may influence pregnancy outcomes, but were not systematically collected in Lotus I and II, are ovarian reserve [42], ovarian stimulation regimens, gonadotropin releasing hormone agonist or antagonist usage, oocyte maturation trigger, and IVF clinical embryology data [43]. Based on the non-availability of such study data, any possible interactions between these factors and luteal phase support remain elusive. However, since the Lotus program enrolled over 2000 patients in 14 countries and was shown to be adequately randomized, it may be assumed that it reflects the general clinical practice of fresh-cycle IVF, where both agonist and antagonist cycles were represented.  Discussion (tracked document, lines: 601-602)  **The rationale for choosing the oral route over the vaginal route is based on patient and physician preference.** Aside from the differences in efficacy between oral dydrogesterone and MVP identified in this study, the administration route of oral dydrogesterone may be advantageous as patients usually prefer to use oral preparations compared to vaginal ones [26, 57, 58]. This may be due to the overall inconvenience [59], administration‑related side effects [14, 15], and cultural barriers associated with using MVP [9]. Although MVP may be the most prescribed route of luteal phase support administration [7], it may not be the most preferred route of administration since women often find vaginal preparations less appealing than oral administration, particularly due to comfort issues [60]. Importantly, improvement in interdisciplinary scientific efforts may be required to increase our understanding of patient needs and preferences [61].  *New text*  The rationale for choosing the oral route over the vaginal route is based on patient and physician preference. Aside from the differences in efficacy between oral dydrogesterone and MVP identified in this study, the administration route of oral dydrogesterone may be advantageous as patients usually prefer to use oral preparations compared to vaginal ones [26, 57, 58]. This may be due to the overall inconvenience [59], administration‑related side effects [14, 15], and cultural barriers associated with using MVP [9]. Although MVP may be the most prescribed route of luteal phase support administration [7], it may not be the most preferred route of administration since women often find vaginal preparations less appealing than oral administration, particularly due to comfort issues [60]. Importantly, improvement in interdisciplinary scientific efforts may be required to increase our understanding of patient needs and preferences [61]. |
| 12 | In the paragraph relating to safety outcomes, explain whether the association of preterm labor and low birth weight with oral progesterone has a rationale and should concern clinicians. | Discussion (tracked document, lines: 507-515)  The safety outcomes included in this meta-analysis of IPD (pooled results analyzed using descriptive statistics) were similar between the oral dydrogesterone and MVP groups in both the maternal and fetus/newborn populations**. Specific complications, such as low birth weight and preterm birth, are known to be associated with assisted reproductive technology. In a 2017 review by Kushnir, et al. [44], low birth rate was reported in 12.7% of infants (6.7% for singletons following single embryo transfer and 7.3% following double-embryo transfer, 56.3% for twins, and 97.4% for higher-order multiples), and the preterm delivery rate was reported as 16.6% (10.5% for singletons, 67.3% for twins, and 92.3% for higher-order multiples).**  *New text*  The safety outcomes included in this meta-analysis of IPD (pooled results analyzed using descriptive statistics) were similar between the oral dydrogesterone and MVP groups in both the maternal and fetus/newborn populations**.** Specific complications, such as low birth weight and preterm birth, are known to be associated with assisted reproductive technology. In a 2017 review by Kushnir, et al. [44], low birth rate was reported in 12.7% of infants (6.7% for singletons following single embryo transfer and 7.3% following double-embryo transfer, 56.3% for twins, and 97.4% for higher-order multiples), and the preterm delivery rate was reported as 16.6% (10.5% for singletons, 67.3% for twins, and 92.3% for higher-order multiples). |
